# Evaluation of fluorometholone as adjunctive medical therapy for trachomatous trichiasis surgery (FLAME): a parallel, double-blind, randomised controlled field trial in the Jimma Zone, Ethiopia

**DOI:** 10.1016/S2214-109X(25)00493-0

**Published:** 2026-01-12

**Authors:** John H Kempen, Aida Abashawl, Ahlam Awad Mohammed, Sarity Dodson, Wondu Alemayehu, Fangming Jin, Alemu Gemechu, Aemero Abateneh Mengesha, Dereje Adugna Kumsa, Yineng Chen, Kathleen McWilliams, Berhanu Tulu, Genemo Abdela, Alemayehu Megersa, Tolossa Cheru, Gadisa Mohammad, Tony Succar, Vatinee Y Bunya, K Davina Frick, Maureen G Maguire, Matthew J Burton, Gui-Shuang Ying, FLAME Trial Research Group

**Affiliations:** **Department of Ophthalmology and Schepens Eye Research Institute, Massachusetts Eye and Ear Infirmary, Boston, MA, USA** (Prof J H Kempen MD, A A Mohammed MPH); **Department of Ophthalmology and Department of Global Health and Social Medicine, Harvard Medical School, Boston, MA, USA** (Prof J H Kempen); **Sight for Souls, Bellevue, WA, USA** (Prof J H Kempen); **MCM Eye Unit, MyungSung Christian Medical Center General Hospital and MyungSung Medical College, Addis Ababa, Ethiopia** (Prof J H Kempen); **Department of Ophthalmology, Addis Ababa University School of Medicine, Addis Ababa, Ethiopia** (Prof J H Kempen); **Berhan Public Health and Eye Care Consultancy, Addis Ababa, Ethiopia** (A Abashawl MD, A A Mohammed, A A Mengesha MD, B Tulu MS, G Abdela MS); **Fred Hollows Foundation, Addis Ababa, Ethiopia and Melbourne, VIC, Australia** (S Dodson DPsych, W Alemayehu MD, A Gemechu MPH, A Megersa MPH, T Cheru MPH, G Mohammad MS); **Scheie Eye Institute and Department of Ophthalmology, Perelman School of Medicine, University of Pennsylvania, Philadelphia, PA, USA** (F Jin MS, Y Chen MS, K McWilliams CCRP, V Y Bunya MD, Prof M G Maguire PhD, Prof G-S Ying PhD); **Department of Ophthalmology, Bahir Dar University College of Medicine and Health Sciences, Bahir Dar, Ethiopia** (A A Mengesha); **Diseases Prevention and Control Directorate, Oromia Regional Health Bureau, Addis Ababa, Ethiopia** (D A Kumsa MPH); **USC Mann School of Pharmacy, Department of Regulatory and Quality Sciences, University of Southern California, Los Angeles, CA, USA** (T Succar PhD); **Department of Ophthalmology, University of Sydney, Sydney, NSW, Australia** (T Succar); **Carey Business School, Johns Hopkins University, Baltimore, ML, USA** (Prof K D Frick PhD); **International Centre for Eye Health, London School of Hygiene & Tropical Medicine, London, UK** (Prof M J Burton PhD); **National Institute for Health Research Biomedical Research, Moorfields Eye Hospital NHS Foundation Trust and University College London Institute of Ophthalmology, London, UK** (Prof M J Burton); **Center for Clinical Epidemiology and Biostatistics, Department of Biostatistics and Epidemiology, Perelman School of Medicine, University of Pennsylvania, Philadelphia, PA, USA** (Prof G-S Ying)

## Abstract

**Background:**

In trachoma, trachomatous trichiasis mediates visual impairment. Trachomatous trichiasis surgery has an unacceptably high relapse incidence. We hypothesised that anti-inflammatory therapy with fluorometholone 0·1% suspension (hereafter fluorometholone) eyedrops perioperatively twice daily for 28 days would safely, efficaciously, and cost-effectively reduce postoperative trachomatous trichiasis relapse (PTT).

**Methods:**

In this randomised, parallel, placebo-controlled trial, we compared placebo (artificial tears) with fluorometholone for eyes undergoing trachomatous trichiasis surgery in participants aged 15 years and older at rural health centres and post sites in the Jimma zone, Ethiopia. Participants were randomly allocated (1:1) to either placebo or fluorometholone. Treatment randomisation was stratified and blocked by surgeon. Participants, surgeons, and outcome assessors were masked to treatment assignment. Outcomes were evaluated at 4 weeks, 6 months, and 12 months postoperatively. The primary outcome was cumulative 12-month incidence of PTT, defined as any of: one or more lashes touching the globe; evidence of epilation; or occurrence of repeat trachomatous trichiasis surgery. This study is registered with ClinicalTrials.gov, NCT04149210.

**Findings:**

Between Aug 19, 2021, and Nov 30, 2024, 2410 participants (1692 [70·2%] self-reported as female and 718 [29·8%] as male; 3235 eyes) were recruited. 1204 participants were randomly allocated to fluorometholone and 1206 to placebo. 823 (34·1%) participants had bilateral trachomatous trichiasis surgery. Baseline treatment group characteristics were similar; 1180 (98·0%) participants in the fluorometholone group and 1181 (97·9%) in the placebo group were evaluated at the 12-month timepoint. In the intention-to-treat analysis, the cumulative incidences of PTT during 12 months of follow-up were 218 (13·4%) of 1625 eyes in the placebo group and 213 (13·4%) of 1593 eyes in the fluorometholone group (95% CI for difference −2% to 2%). Prespecified secondary efficacy and safety outcomes were not statistically different between groups (all p≥0·10). The incidence of adverse events attributed to study treatment was nine (0·7%) in the placebo group and four (0·3%) in the fluorometholone group (p=0·17); satisfaction with surgery (satisfied or very satisfied) was recorded for 3156 (99·7%) of 3167 eyes. Health economic considerations did not favour programmatic use of fluorometholone given its inefficacy.

**Interpretation:**

Fluorometholone twice daily for 4 weeks was safe but not efficacious or cost-effective for reducing PTT, and hence is not recommended for programmatic use.

**Funding:**

National Eye Institute (National Institutes of Health) and AbbVie.

## Introduction

Trachoma, a neglected tropical disease, is the leading infectious cause of blindness worldwide.^1-4^ Trachomatous trichiasis, a sequela of conjunctival scarring resulting from repeated *Chlamydia trachomatis* infection and chronic inflammation, causes severe chronic eye pain and is the primary pathway to trachomatous visual impairment. Approximately 1·9 million people worldwide are vision impaired from trachoma, causing 1·4% of blindness;^5^ about 1·5 million people had untreated trachomatous trichiasis and were at risk of blindness as of April 15, 2024.^4^ Ethiopia has the largest number of cases of trachoma worldwide.^4^

Surgery to correct trichiasis is one of the four WHO-endorsed priority interventions for programmes aiming to prevent trachoma blindness.^2,3^ Ethiopia is currently implementing the WHO-endorsed programme in accordance with WHO recommendations to alleviate its burden of trachoma. A high recurrence rate following trichiasis surgery reduces the benefits of surgery and undercuts community confidence, resulting in less use by people who could benefit from surgery. A review of recurrent trachomatous trichiasis (postoperative trachomatous trichiasis [PTT]) for the currently WHO-recommended procedures (bilamellar tarsal rotation and posterior lamellar tarsal rotation) reported a PTT incidence of 9–33%.^6^ PTT is a difficult problem with worse outcomes of surgical repair than primary trachomatous trichiasis.^7^

Ongoing conjunctival inflammation in trachoma-endemic settings is associated with progressive or contractile scarring,^8,9^ and often is seen in individuals with trachomatous trichiasis.^10,11^ Such inflammation is only rarely associated with active *C trachomatis* infection,^11,12^ and the specific causes thereof are incompletely understood. In the STAR trial, postoperative azithromycin therapy was associated with a reduction risk of 3·4 PTT events per 100 person-years even though detectable *C trachomatis* infection was rare; anti-inflammatory effects of azithromycin were cited as one potential mechanism by which benefit might have occurred.^13^

Fluorometholone 0·1% suspension (hereafter fluorometholone) is a low-potency topical corticosteroid used for treating episcleritis,^14^ with inflammation at a similar level of the conjunctiva to that in trachoma. To test the hypothesis that perioperative topical anti-inflammatory therapy might be safe and effective for reducing the incidence of PTT, we conducted a preliminary trial involving 155 participants, comparing three alternative doses of fluorometholone with placebo-treated and untreated second eyes, finding that all three doses were associated with an approximately one-third reduction in PTT with minimal side effects.^15^ Motivated by the promising treatment effect from this preliminary trial, we conducted a definitive, large-scale trial for comparing the lowest dose—twice-daily fluorometholone for 28 days—with placebo (artificial tears) as adjunctive therapy for trachomatous trichiasis surgery in the programmatic setting. Specific aims of the study were: to assess the efficacy of one drop of fluorometholone twice daily for 4 weeks in reducing the incidence of PTT when given as adjunctive therapy with trachomatous trichiasis surgery in the programmatic setting; to assess whether such treatment is sufficiently safe for wide-scale implementation in trachomatous trichiasis programmes; and to estimate the costs of adding fluorometholone treatment to trachomatous trichiasis surgery per case of PTT averted, and to characterise the value of such treatment under a range of plausible health economic circumstances. Here we report the primary results from this trial.

## Methods

### Study design and participants

The Fluorometholone as Adjunctive Medical Therapy for Trachomatous Trichiasis Surgery (FLAME) trial was a randomised, masked (participant, graders, lead investigators), parallel-group, placebo-controlled field trial in the rural Jimma zone of Ethiopia between Aug 19, 2021, and Nov 30, 2024. A detailed report of the design of the trial has been published previously,^16^ and the final version of the study protocol is available online.

Potential participants were mobilised from the community to trachomatous trichiasis surgery campaign events typically conducted at a health centre or post in rural parts of Ethiopia. From this screening population of individuals undergoing upper-lid trachomatous trichiasis surgery who did not meet exclusion criteria, consenting adults aged 18 years and older—or children aged 15–17 years with parental consent and child assent—were randomly allocated (1:1) to fluorometholone or placebo. The random allocation to either placebo or fluorometholone was done at the participant level. Exclusion criteria are summarised in the protocol; in brief, these included contraindications to study treatments, medical conditions that would interfere with standard trachomatous trichiasis surgery management or follow-up, and PTT in all potential study eyes. Potential participants with PTT in one eye, and previously unoperated (primary) trachomatous trichiasis in the other eye, could be enrolled with only the primary trachomatous trichiasis eye as a study eye. Baseline data were collected before randomisation. Age, sex, and ethnicity data were self-reported by participants. All eyes operated on were included in the trial; when two eyes were included, both received the same treatment. Follow-up was done at the same or at a different but similar, convenient location.

The study was implemented to evaluate what effect the addition of ancillary treatment with fluorometholone might have on existing programmes. Therefore, we conducted the trial within the context of an unaltered ongoing trachomatous trichiasis programme in remote, rural parts of the Jimma zone, Oromia, Ethiopia—an area known to have a high burden of trachomatous trichiasis.^17^ Mobilisation and surgery were carried out as per the norms for the programme, collaboratively led by the Jimma zonal branch of the Oromia Regional Health Bureau and the Fred Hollows Foundation (the study’s surgical team; Sydney, NSW, Australia). Qualified trachomatous trichiasis surgeons for the programme (and thus the study) were recruited by the surgical teams from local areas where the outreach was taking place, an approach similar to that likely to be used over the years to come. In Ethiopia, and in nearly all trachoma-endemic countries, trachomatous trichiasis surgery is done by specially trained nurses or optometrists, selected based on exceptional skill. Surgeons continued to practise the surgical technique they were most comfortable with between bilamellar tarsal rotation (BLTR) and posterior lamellar tarsal rotation (PLTR), in accordance with WHO guidance.^18^ Ultimately, 30 trachomatous trichiasis surgeons (recruited per programmatic routine from the local vicinity where trachomatous trichiasis surgery was to take place) participated in the trial, seven performing BLTR and 23 performing PLTR. Consistent with the WHO guidance that new surgeons should be trained in PLTR,^18^ the BLTR surgeons were more experienced than the PLTR surgeons (performing twice as many surgeries per year and having 13 times more years of experience).^19^ Consistent with the overall philosophy of not changing the trachomatous trichiasis programme, potential variation between surgeons, surgical techniques, and region of the participant’s residence were addressed by stratifying randomisation by surgeon rather than by altering surgeon practices; surgeons were recruited and received training or refresher training per programmatic routine (appendix 3 p 1). The programme uniformly used dissolvable sutures.

The FLAME trial has been implemented in accordance with the Declaration of Helsinki and under institutional review board approval by the Ethiopian National Research Ethics Review Board, the Ethiopian Food and Drug Administration, the Mass General Brigham (Boston, MA, USA) institutional review board (the single institutional review board for US sites), and the institutional review board of the London School of Hygiene & Tropical Medicine (London, UK). A letter of support from the Oromia Regional Health Bureau was also obtained as required. The study is registered at ClinicalTrials.gov, NCT04149210. A data and safety monitoring committee convened by the National Eye Institute (the funding agency) also monitored the study to ensure the safety of participants and robust implementation of the protocol.

### Randomisation and masking

Randomisation and masking are described in more detail in the FLAME design paper.^16^ In brief, a surgeon-stratified randomisation sequence with block sizes of two or four was prepared by a masked biostatistician of the Data Center in advance of the study. Masking was applied to participants, surgeons, research field team members, and most senior investigators, including the study chair, vice-chair, and Data Center Director. The Deputy Director of the Data Center, master-level statistician, and data and safety monitoring committee were unmasked. Masking discipline was maintained until data collection was completed and the database locked. Masking was accomplished by repackaging the active study drug (fluorometholone as brand name FML [Allergan, Dublin, Ireland]) and placebo (Soothe XP artificial tears [Bausch & Lomb, Vaughan, ON, Canada]) into identical bottles, which was done after confirming the potency of each drug batch at the University of Pennsylvania Investigational Drug Service (Philadelphia, PA, USA) using a sterile technique under a hood. Repackaged investigational drug product bottles were used to create numbered medication kits, each of which was associated with a study identifier number in the pre-prepared randomisation sequence. Batches of 20 medication kits were inserted into boxes in the randomisation sequence order. Boxes of 20 were used by field teams to give the assigned medication kit to each surgeon’s enrolled participants until the box was depleted, thus accomplishing stratification for surgeon (which also accomplished stratification for the type of surgery performed and for the geographic location). The study drug was transported and stored with temperature control within the range specified for both active treatment and placebo, using the more restrictive range of the placebo (15–25°C). The donated drug used was found by the Investigational Drug Service to be within expectations in terms of potency, and studies of the potency over time showed that greater than 90% potency was maintained for 1 year. Any investigational drug product unused within 12 months of repackaging was destroyed.

Study eyes (one or two eligible eyes per participant in the same treatment group) received the first drop before surgery, which was continued twice daily after surgery for a total of 28 days and then stopped. Adherence was assessed by a diary, questionnaire, and bottle weight with a scale able to detect a difference in weight corresponding to a single eyedrop. These tools were used to define two adherence levels approximately bracketing half of the distribution (>75% of perfect adherence and ≤75% of perfect adherence) following the following algorithm (see statistical analysis plan): if the bottle weight change is known and there is 75% or less of expected doses, then the participant has less than 75% adherence; if the bottle weight change is greater than 75% of the expected doses, then the participant has more than 75% adherence; if the medication diary indicates more than 75% and the bottle weight change is unknown, then the participant has more than 75% adherence; if the bottle weight is unknown and the medication diary is 75% or less, then the participant has less than 75% adherence; and if the medication diary is unknown and the bottle weight is unknown, use the self-report adherence and only classify participants with self-reported compliance of Very Good as more than 75% adherence.

### Procedures

Study participants were seen and data collected at the time of enrolment (visit 1), at the time of surgery (visit 2), and then for three follow-up visits at 28 days (within 4 days; visit 3), 6 months (within 60 days; visit 4), and 1 year (within 90 days; visit 5) after trachomatous trichiasis surgery. The scope of data collected at these visits is summarised in the design paper,^16^ addressing outcomes of surgery (both efficacy and safety outcomes), demographic and clinical characteristics, participant-reported outcome measures, and adherence to treatment.

### Outcomes

The primary outcome was the cumulative incidence of PTT by 12 months, defined as at least one of the following: one or more lashes touching the globe in an eye; evidence of epilation on clinical examination; or occurrence of repeat trachomatous trichiasis surgery. Examination data were collected by trained teams each consisting of a study nurse and a data enterer. The examining nurse conducted data collection requiring clinical skills, including counting of aberrant eyelashes and signs of epilation and other clinical grading, using 2·5 times magnifying loupes and a flashlight. Presenting visual acuity was measured using the Peek Acuity application (Peek Vision, London, UK),^20^ and intraocular pressure was measured as the average of three measurements using an iCARE 200 tonometer (Vantaa, Finland). Visual acuity was also transformed into the logarithm of the minimal angle of resolution (logMAR) multiplied by −1 (logMAR=[−log_10_(visual acuity fraction)]), a transformation in which lower variables are more favourable and each step of 0·1 corresponds to one line on a standard visual acuity chart.^21^ Key outcome measurements were regularly compared with measurement by a study field supervisor expert optometrist (GA) to monitor for and avoid drift in measurements and gradings over time. The data were entered into a custom REDCap (Nashville, TN, USA) mobile app database with range, consistency, and completeness checks. De-identified data entered in the field were transmitted automatically over a cellular network (live or later, when a network was available) to the Data Center at the University of Pennsylvania, which monitored the data completeness and quality throughout the study. Additional quality control strategies have been described for the FLAME trial previously.^16^ Data collection was done in the Jimma zone, Ethiopia. COVID-19 avoidance strategies were implemented throughout the trial.

### Statistical analysis

The study sample size calculation to determine how many participants to enrol is detailed in the design paper^16^ and statistical analysis plan. In brief, the initial sample size was 2254 participants (3944 study eyes), assuming PTT by 12 months of 20% in the placebo group and 15% in the fluorometholone group (a 25% reduction in PTT rate), 75% of participants having had bilateral trachomatous trichiasis surgery, an inter-eye correlation coefficient of 0·48 in PTT, and 10% of participants lost to follow-up. The initial sample size was based on 90% power with a type I error rate of 5%. During the interim analysis results from cumulated masked data by Oct 26, 2022, when 932 participants were enrolled and 508 had completed the study, the sample size was recalculated based on a lower than anticipated bilaterality of trachomatous trichiasis surgery (updated from 75% to 35%) and slightly lower than anticipated PTT incidence (updated from 20% to 19%) and inter-eye correlation (updated from 0·48 to 0·46). The final sample size of approximately 2400 participants (3240 study eyes), adopted with permission from the data and safety monitoring committee, provided 90% power to detect a hypothesised clinically relevant 25% reduction (from 19·0% to 14·3%) in PTT.

The final statistical analysis plan also lists all variable specifications. In brief, the baseline demographic and ocular characteristics by treatment group were summarised using mean (SD) for continuous measures and n (%) for categorical measures. For the comparison of the primary outcome (cumulative incidence rate of PTT by 12 months) between the two treatment groups, repeated-measures logistic regression models were used to account for the inter-eye correlation in participants with trachomatous trichiasis surgery in both eyes through generalised estimating equations, and the surgeon (the stratified randomisation factor) was included as a covariate. The difference between the two treatment groups in the cumulative incidence rate of PTT by 12 months, the odds ratio (OR), and 95% CIs were calculated from the repeated measures logistic regression model. Similar analyses were done separately for comparison of PTT at each timepoint (4 weeks, 6 months, and 12 months), and for incident PTT in the first 6 months and after 6 months, between the two treatment groups. All analyses were done with participants’ randomised treatment group assignment regardless of their treatment adherence (intention-to-treat population). Because very few data were missing (approximately 2% for the primary outcome), no imputations were carried out.

To check the consistency of results across subgroups, prespecified subgroup analyses were done by baseline upper-eyelid trichiasis severity (severe or not severe), baseline conjunctival inflammation (presence or absence), and treatment adherence (>75% or ≤75%; appendix 3 p 2). The difference between subgroups was assessed by testing for interaction in the repeated logistic regression model that included the treatment group indicator, subgroup indicator, their interaction term, and the surgeon as a covariate. For the statistical comparison of these eye-specific secondary outcomes and safety outcomes between treatment groups, the generalised linear model through generalised estimating equations was applied to account for the inter-eye correlation, and a binomial model was used for the binary outcome (eg, the incidence of PTT), a Poisson model for the count outcome (number of lashes touching the globe), and a linear regression model for the continuous outcome (eg, pain score in study eyes at 1 year). When possible (eg, when there was sufficient event count for reliable statistical modelling), the effect of the stratification factor (surgeon) on secondary outcomes and safety outcomes was accounted for by including the surgeon as a covariate. When the number of events was too small for statistical modelling (16 or less in each group), statistical comparisons for risk difference were based on Fisher’s exact test and exact 95% CIs. All statistical analyses were done in SAS (version 9.4) and two-sided p less than 0·05 was considered statistically significant.

### Role of the funding source

Representatives of the National Eye Institute (the primary funder) participated in study governance activities as per National Institutes of Health principles for cooperative agreement awards. Otherwise, none of the funders were involved in study design, data collection, data analysis, data interpretation, writing of the report, or the decision to submit for publication.

## Results

From Aug 19, 2021, to Nov 8, 2023, 2663 individuals presenting for programmatic trachomatous trichiasis surgery were evaluated for study participation and 2410 (90·5%; 3235 eyes undergoing trachomatous trichiasis surgery; figure) were enrolled as study participants. Those not participating in the study also had trachomatous trichiasis surgery performed at study expense if they wished. Non-enrolment was primarily on the basis of ineligibility (meeting exclusion criteria).

1206 participants (1632 eyes) were randomly assigned to the placebo group and 1204 participants (1603 eyes) to the fluorometholone group. Person-level and eye-level (table 1) characteristics of the two groups were well balanced, including self-reported age and sex, and trachomatous trichiasis severity measures such as extent of upper eyelid trichiasis and of upper eyelid entropion. All participants self-reported African ancestry. Among enrolled participants, the mean age at baseline in both treatment groups was approximately 48 years (SD 15), with a median of 45 years (IQR 35–60) in the placebo group and 50 years (35–60) in the fluorometholone group. 856 (71·0%) participants were female and 350 (29·0%) male in the placebo group, and 836 (69·4%) were female and 368 (30·6%) male in the fluorometholone group. At enrolment, 425 (35·2%) participants in the placebo group and 398 (33·1%) participants in the fluorometholone group underwent bilateral trachomatous trichiasis surgery. Baseline mean intraocular pressure was 12·0 mm Hg (SD 3·2) in the placebo group and 11·9 mm Hg (3·2) in the fluorometholone group. Cataract diagnosis at baseline was in 18 (1·1%) eyes in the placebo group and 26 (1·6%) in the fluorometholone group. Among 312 eyes that were graded by the study supervisor, the inter-rater agreements between the study supervisor and study nurses were very high, with agreement in the range of 96·7–100% and κ ranging from 0·92 to 1·00, suggesting high consistency in determination of primary outcome.

12-month follow-up was completed for 1600 (98·0%) eyes of 1181 (97·9%) participants in the placebo group and 1567 (97·8%) eyes of 1180 (98·0%) participants in the fluorometholone group. An additional 20 participants (25 eyes) in the placebo group and 17 participants (26 eyes) in the fluorometholone group did not complete 12-month follow-up but contributed 4-week or 6-month data to the primary analysis for cumulative incidence of PTT. The major reasons for incomplete follow-up were death (14 [0·6%] participants) and loss to follow-up (28 [1·2%] participants) from being repeatedly away from the area, eg, from migrating (figure).

The primary study outcome of PTT incidence (table 2, appendix 3 p 4) noted at some point during the 1 year of follow-up occurred in 218 (13·4%) eyes in the placebo group and 213 (13·4%) in the fluorometholone group (OR 1·00 [95% CI 0·80 to 1·25], absolute risk difference 0·00 [95% CI −0·02 to 0·02]). In both treatment groups, PTT occurred more often within the first 6 months (9·2% incidence rate in both treatment groups) than the second 6 months of follow-up (4·3% in both groups). Prespecified subgroup analyses showed that between-treatment differences were similar by baseline upper eyelid trichiasis severity (p=0·50), baseline conjunctival inflammation (p=0·53), and treatment adherence (p=0·21). Non-prespecified analysis of potential interaction with sex also found no interaction (p=0·24; appendix 3 p 3).

Prespecified secondary clinical outcomes reflecting submorphologies of PTT were also similarly distributed across treatment groups, with no statistically significant differences between groups (table 3). Safety outcomes of trachomatous trichiasis surgery or treatment also are summarised in table 3. The distribution of corneal opacity at 12 months was similar in the placebo group (532 [33·2%] study eyes) and fluorometholone group (503 [32·1%] study eyes); each was slightly less than it had been at baseline (586 [35·9%] in the placebo group and 599 [37·4%] in the fluorometholone group; tables 1 and 3). The mean improvement in visual acuity from baseline to 12 months was similar at 0·02 logMAR (SD 0·36) in the placebo group and 0·03 logMAR (SD 0·33) in the fluorometholone group, which corresponded to a 1 or 1·5 letter improvement on a standardised visual acuity chart. Prespecified safety outcomes related to trachomatous trichiasis surgery (overcorrection, eyelid contour abnormality including notching, lid closure defect, and granuloma) were based on findings at the 1-year follow-up. These outcomes were all uncommon (≤3·6%) by 1 year in both treatment groups without statistically significant differences (all p≥0·17). Infections of the cornea, conjunctiva, or other sites were uncommon in both groups, diagnosed by study nurses in 38 (2·35%) study eyes in the placebo group versus 24 (1·52%) in the fluorometholone group at 4 weeks (p=0·14), four (0·25%) versus three (0·19%) at 6 months (p=0·72), and four (0·25%) versus five (0·32%) at 12 months (p=0·76). Intraocular pressure elevation change from baseline by 10 mm Hg or more was similarly rare in both groups, at five (0·3%) study eyes in the placebo group versus seven (0·4%) in the fluorometholone group at week 4, and 16 (1·0%) in each group within 12 months. Cataract surgery had been performed for seven (0·4%) study eyes in the placebo group versus three (0·2%) in the fluorometholone group by 1 year. The overall incidence of adverse events attributed to the study treatment determined by the masked observers was nine (0·7%) study eyes in the placebo group and four (0·3%) in the fluorometholone group.

In terms of participant-reported outcomes at 12-month follow-up (table 4), participants reported a high degree of overall and cosmetic satisfaction with their outcome of trachomatous trichiasis surgery, with more than 99% very satisfied or satisfied in both groups. Overall satisfaction with surgery was slightly less favourable with placebo than fluorometholone (p=0·04), with 1461 (91·3%) study eyes in the placebo group and 1468 (93·7%) in the fluorometholone group reporting being very satisfied. Satisfaction with the cosmetic outcome was similar in the two groups (p=0·12), with 1458 (91·1%) study eyes in the placebo group and 1457 (93·0%) in the fluorometholone reporting being very satisfied. The distribution of EuroQol EQ-5D-measured health utility was similarly favourable in the two groups, with mean health state scores of 80·2 (SD 15·5) in the placebo group and 79·6 (15·7) in the fluorometholone group (p=0·28).

For the primary health economic analysis, because the active treatment did not reduce the number of cases of PTT and administering drops costs more than not administering them, the health economic properties are unfavourable with fluorometholone.

## Discussion

This large-scale field trial for trachomatous trichiasis surgery in the programmatic setting found that fluorometholone eyedrops given for 28 days perioperatively as an adjunctive therapy did not reduce risk of PTT. Low efficacy, and in turn a less favourable health economic profile, suggest that fluorometholone is not a suitable addition to trachomatous trichiasis programmes. Given that our previous study found similar results for higher doses or longer duration of treatment to those with the fluorometholone dose studied here, it is unlikely that higher doses would be more effective. The most likely explanation for the apparent discrepancy between our preliminary trial results—which suggested a benefit of fluorometholone—and the negative results of the definitive FLAME trial is that the former results were observed by chance; the apparent benefit did not reach statistical significance, being a phase 2-like trial designed to show potential effectiveness in relation to doses rather than to prove efficacy. It is unlikely that the use of BLTR surgery exclusively in the preliminary trial could explain the difference given that no benefit was seen in the FLAME trial among participants operated on using BLTR (one-third of surgeries in the FLAME trial). It also seems unlikely that possibly increased adherence to treatment in the preliminary trial could have led to so much larger results without any suggestion of an efficacy signal in the FLAME trial. For most medications, including intraocular pressure-lowering eyedrops for glaucoma, patients adhere to therapy to a lesser degree than the participants in the FLAME Trial did, and efforts to increase adherence improve adherence to a level less than 75%;^22^ therefore, it is unlikely that greater adherence than observed in the FLAME trial could be achieved in implementation programmes. Likewise, it is unlikely that fluorometholone works better with greater trachomatous trichiasis severity (as in the preliminary trial), given the absence of a treatment–severity interaction in the FLAME trial.

Taken together with a previous trial indicating that the anti-inflammatory and antifibrotic properties of doxycycline were not associated with a reduction in PTT,^9^ and no replication of the STAR trial results with azithromycin (an agent with anti-inflammatory effects) in another trial,^23^ it appears unlikely that anti-inflammatory therapy will be a useful adjunct for trachomatous trichiasis surgery programmes. Although fluorometholone proved unsuccessful, recent publications suggest that potentially large improvements in PTT incidence can be obtained with use of PLTR versus BLTR surgery (at least for new surgeons),^19,24-26^ and with use of refresher training using a mannequin and supportive supervision for established trachomatous trichiasis surgeons.^27^

In terms of safety, one drop of fluorometholone twice daily for 4 weeks had a risk of corticosteroid class side-effects similar to placebo, with less than 0·5% incidence of intraocular pressure elevation at 4 weeks and less than 0·5% incidence of cataract surgery by 1 year. These safety results in a very large, prospectively observed cohort suggest that fluorometholone is safe when used for short-term treatment for various indications.

The study also found that the results of trachomatous trichiasis surgery in this programmatic context were favourable, with only 13·4% of study eyes developing PTT within 12 months and 99% reporting being satisfied or very satisfied with surgical outcomes. These results support the value of ongoing conduct of trachomatous trichiasis surgery in programmatic contexts similar to the one studied, in which 30 surgeons deployed from the local area were used rather than restricting study surgery to a highly selected group of the best surgeons who could be found. Such programmes should be feasible as long as supportive funds can be found.

Limitations of the study include a lower PTT incidence than assumed in the initial and subsequent sample size calculations. The PTT incidence declined as the study went on, in association with greater use of PLTR and less use of BLTR over time, the former having better outcomes in our study. However, the 95% CI on the PTT rate difference covered 2% more or less than placebo with the fluorometholone treatment, meeting our detectable difference objectives. Thus, the trial as implemented was sufficiently powered to achieve the study design objective. Adherence to the study treatment based on a hierarchy of adherence metrics (bottle weight change, medication diary, self-reported adherence), giving priority to bottle weight change (which was missing for about 25% of participants), found slightly more than half of participants at more than 75% of perfect treatment adherence. The subgroup analysis among participants with more than 75% of ideal treatment adherence also did not find a significant reduction in PTT (12·9% in the placebo group *vs* 11·9% in the fluorometholone group). Given that in a programmatic context it is unlikely that adherence would have been better than in our clinical trial, it is unlikely that programmatic impact of fluorometholone would differ qualitatively from the absence of effect observed in the FLAME trial; it is also unlikely that no signal would be seen with this level of adherence if fluorometholone were effective.

In conclusion, perioperative fluorometholone 0·1% suspension administered immediately preceding and twice daily following trachomatous trichiasis surgery for 28 days did not affect the incidence of PTT, but had a favourable safety profile that could be generalisable to a wide range of indications for anti-inflammatory therapy. More than 90% of participants were very satisfied with the results of trachomatous trichiasis surgery, supporting the value of trachomatous trichiasis surgery programmes to reduce avoidable blindness and visual impairment as a result of this neglected tropical disease of poverty.

## Supplementary Material

1

2

3

## Figures and Tables

**Figure:**
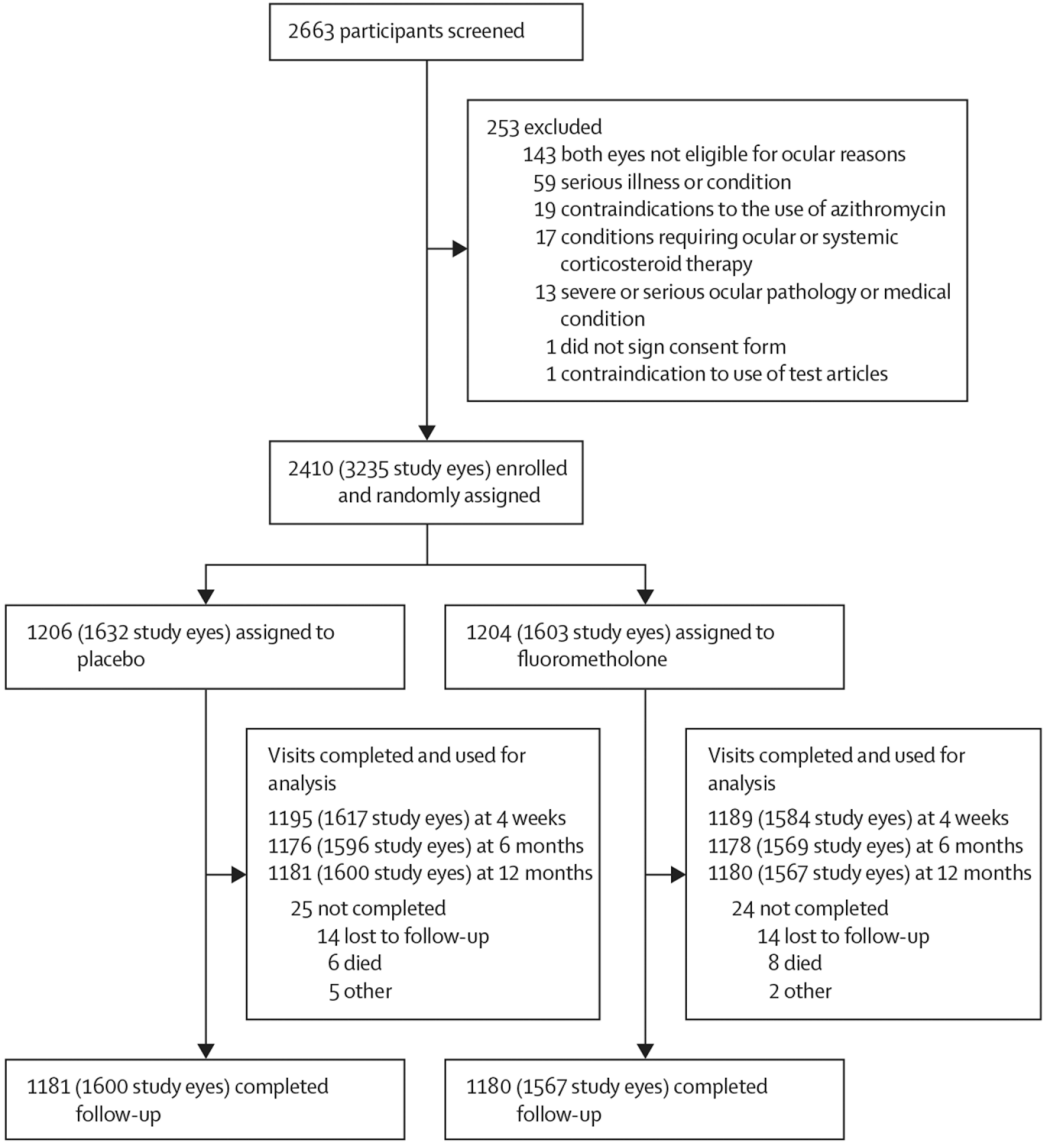
Trial profile

**Table 1:** Baseline participant and study eye characteristics by treatment group

	Placebo (n=1206 participants, 1632 study eyes)	Fluorometholone (n=1204 participants, 1603 study eyes)
Age, years	47·8 (15·2)	47·3 (14·9)
Sex		
Female	856 (71·0%)	836 (69·4%)
Male	350 (29·0%)	368 (30·6%)
Laterality of trachomatous trichiasis surgery		
Unilateral	779 (64·6%)	803 (66·7%)
Bilateral	425 (35·2%)	398 (33·1%)
Reading ability: unable	1085 (90·0%)	1082 (89·9%)
Self-reported pain or discomfort		
None	335 (27·8%)	316 (26·2%)
Slight	418 (34·7%)	419 (34·8%)
Moderate	234 (19·4%)	249 (20·7%)
Severe	182 (15·1%)	169 (14·0%)
Extreme	37 (3·1%)	51 (4·2%)
Ocular Surface Disease Index ocular symptoms score	48·6 (29·5)	48·5 (29·9)
Ocular Surface Disease Index total score	37·8 (23·2)	37·9 (23·4)
Type of trachomatous trichiasis surgery*		
Bilamellar tarsal rotation	524 (32·1%)	515 (32·1%)
Posterior lamellar tarsal rotation	1105 (67·7%)	1084 (67·6%)
No surgery due to withdrawal after randomisation	3 (0·2%)	4 (0·2%)
Presenting visual acuity		
20/20	103 (6·3%)	81 (5·1%)
20/25–20/40	586 (35·9%)	566 (35·3%)
20/50–20/63	438 (26·8%)	466 (29·1%)
20/80–20/160	344 (21·1%)	321 (20·0%)
20/200–20/400	88 (5·4%)	92 (5·7%)
Worse than 20/400	73 (4·5%)	77 (4·8%)
Total number of upper eyelid lashes touching the globe or the cornea		
Mean	3·9 (4·8)	3·8 (4·8)
0 (epilating)	236 (14·5%)	252 (15·7%)
1–5	1058 (64·8%)	1005 (62·7%)
6–9	143 (8·8%)	178 (11·1%)
10–19	152 (9·3%)	130 (8·1%)
≥20	43 (2·6%)	38 (2·4%)
Evidence or extent of epilation in the upper eyelid		
None	1386 (84·9%)	1341 (83·7%)
<1/3 of the lid margin	39 (2·4%)	30 (1·9%)
≥1/3 to ≤2/3 of the lid margin	75 (4·6%)	81 (5·1%)
>2/3 of the lid margin	132 (8·1%)	151 (9·4%)
Severity of upper eyelid trichiasis		
Severe: total number of lashes ≥6, or epilation ≥1/3	541 (33·1%)	576 (35·9%)
Not severe	1091 (66·9%)	1027 (64·1%)
Upper eyelid entropion		
E0: None	176 (10·8%)	168 (10·5%)
E1: Mild	642 (39·3%)	598 (37·3%)
E2: Moderate	466 (28·6%)	439 (27·4%)
E3: Severe	246 (15·1%)	287 (17·9%)
E4: Total	102 (6·3%)	111 (6·9%)
Conjunctivalisation of the lid margin grade		
CM0: None	25 (1·5%)	16 (1·0%)
CM1: posterior to the line of meibomian gland	169 (10·4%)	175 (10·9%)
CM2: less than 50% of the lid	627 (38·4%)	589 (36·7%)
CM3: greater than 50% of the lid	811 (49·7%)	823 (51·3%)
Trichiasis of the lower eyelid†		
No	1571 (96·3%)	1509 (94·1%)
Yes	61 (3·7%)	94 (5·9%)
Corneal opacity		
C0: None	1046 (64·1%)	1004 (62·6%)
C1: Peripheral	357 (21·9%)	376 (23·5%)
C2a: Off centre faint	75 (4·6%)	79 (4·9%)
C2b: Off centre dense	16 (1·0%)	12 (0·7%)
C2c: Central faint	110 (6·7%)	95 (5·9%)
C2d: Central dense	18 (1·1%)	24 (1·5%)
C3: Total and central dense	10 (0·6%)	13 (0·8%)
C4: Phthisis	0	0
Upper eyelid conjunctival scarring		
S0: None	86 (5·3%)	69 (4·3%)
S1: Mild	90 (5·5%)	102 (6·4%)
S2: Moderate	806 (49·4%)	811 (50·6%)
S3: Severe	650 (39·8%)	621 (38·7%)
Intraocular pressure, mm Hg	12·0 (3·2)	11·9 (3·2)
Diagnosed with cataracts: Yes	18 (1·1%)	26 (1·6%)
Previous eye surgery: Yes	7 (0·4%)	7 (0·4%)
Previous eye injury: Yes	23 (1·4%)	27 (1·7%)

Data are mean (SD) or n (%). For participant characteristics n denotes number of participants, and for ocular characteristics number of study eyes. *Stratification by surgeon accomplished stratification of use of bilamellar or posterior lamellar tarsal rotation. †Trachomatous trichiasis is defined as one or more eyelashes touching the eye, or evidence of epilation.

**Table 2:** Comparison of primary outcome (PTT) between treatment groups

	Placebo (n=1625 study eyes)	Fluorometholone (n=1593 study eyes)	Adjusted risk difference*	Adjusted OR*	p value*
PTT at any time during 1-year follow-up	218/1625 (13·4%)	213/1593 (13·4%)	0·00 (−0·02 to 0·02)	1·00 (0·80 to 1·25)	0·97
PTT present at 4 weeks	32/1617 (2·0%)	36/1584 (2·3%)	0·00 (−0·01 to 0·01)	1·13 (0·68 to 1·90)	0·63
PTT present at 6 months	137/1596 (8·6%)	136/1569 (8·7%)	0·00 (−0·02 to 0·02)	1·01 (0·78 to 1·33)	0·92
PTT present at 12 months	168/1600 (10·5%)	167/1567 (10·7%)	0·00 (−0·02 to 0·02)	1·02 (0·80 to 1·32)	0·85
Early-onset PTT (incident by the time of the 6-month visit)	150/1623 (9·2%)	146/1593 (9·2%)	0·00 (−0·02 to 0·02)	0·99 (0·76 to 1·28)	0·92
Late-onset PTT (incident after the 6-month visit)	68/1600 (4·3%)	67/1567 (4·3%)	0·00 (−0·01 to 0·01)	1·01 (0·70 to 1·46)	0·95
Baseline upper eyelid trichiasis severity†					
Severe‡	105/538 (19·5%)	100/573 (17·5%)	−0·01 (−0·06 to 0·03)	0·90 (0·64 to 1·27)	0·50§
Not severe	113/1087 (10·4%)	113/1020 (11·1%)	0·00 (−0·02 to 0·03)	1·05 (0·78 to 1·42)	··
Baseline conjunctival inflammation†					
Presence	33/149 (22·1%)	38/194 (19·6%)	−0·03 (−0·12 to 0·06)	0·82 (0·46 to 1·47)	0·53§
Absence	185/1476 (12·5%)	175/1399 (12·5%)	0·00 (−0·02 to 0·03)	1·01 (0·79 to 1·28)	··
Treatment adherence†¶					
>75%	104/807 (12·9%)	105/883 (11·9%)	−0·02 (−0·05 to 0·02)	0·86 (0·63 to 1·17)	0·21§
≤75%	113/808 (14·0%)	106/698 (15·2%)	0·02 (−0·02 to 0·05)	1·15 (0·83 to 1·59)	··

Data are n/N (%), adjusted risk difference (95% CI), adjusted OR (95% CI), or p value. OR=odds ratio. PTT=postoperative trachomatous trichiasis. *From generalised regression models that account for inter-eye correlation and are adjusted by the surgeon (the stratification factor randomisation), using the placebo group as the reference group. †Subgroup analyses were done by calculating the cumulative 1-year incidence rate of PTT in the placebo group and fluorometholone group, the adjusted difference between the two treatment groups and adjusted OR using the placebo group as the reference in each subgroup, and the p value for testing of interaction between the treatment group indicator and subgroup indicator. ‡Severe was defined as total number of lashes of six or more, or epilation of one-third or more. §Interaction p value. ¶Ten eyes in the placebo group and 12 eyes in the fluorometholone group were excluded from the analysis due to missing data on treatment adherence.

**Table 3:** Comparison of secondary efficacy outcomes and safety outcomes between treatment groups

	Placebo (n=1625 study eyes)	Fluorometholone (n=1593 study eyes)	Adjusted risk difference (95% CI)	p value
Secondary efficacy outcome				
Reoperation for PTT during 1-year follow-up	1 (0·1%)	3 (0·2%)	0·00 (−0·00 to 0·00)*	0·37†
Number of upper eyelid lashes touching the cornea at 1 year				
Mean	0·16 (0·73)	0·13 (0·57)	−0·02 (−0·06 to 0·01)‡	0·23‡
No trichiasis	1466 (91·6%)	1446 (92·3%)	··	··
Zero (epilating)	8 (0·5%)	8 (0·5%)	··	··
1–5	121 (7·6%)	110 (7·0%)	··	··
≥6	5 (0·3%)	3 (0·2%)	··	··
Total number of upper eyelid lashes touching the globe or the cornea at 1 year				
Mean	0·22 (0·86)	0·21 (0·78)	−0·01 (−0·05 to 0·04)‡	0·75‡
No trichiasis	1433 (89·6%)	1402 (89·5%)	··	··
Zero (epilating)	8 (0·5%)	8 (0·5%)	··	··
1–5	150 (9·4%)	151 (9·6%)	··	··
≥6	9 (0·6%)	6 (0·4%)	··	··
Presence of entropion in the upper eyelid at 1 year	208 (13·0%)	234 (14·9%)	0·02 (−0·01 to 0·04)‡	0·13‡
Severity among eyes with entropion				
Mild	176 (84·6%)	201 (85·9%)	··	··
Moderate	27 (13·0%)	22 (9·4%)	··	··
Severe	5 (2·4%)	10 (4·3%)	··	··
Complete	0	1 (0·4%)	··	··
Visual acuity at 1 year				
Mean in logMAR	0·48 (0·48)	0·48 (0·48)	−0·01 (−0·04 to 0·03)‡	0·78‡
Mean change from baseline in logMAR	−0·02 (0·36)	−0·03 (0·33)	−0·01 (−0·04 to 0·01)‡	0·36‡
20/200 or worse	135 (8·4%)	117 (7·5%)	−0·01 (−0·03 to 0·01)‡	0·30‡
Safety outcomes				
Corneal opacity in study eye at 1 year	··	··	··	0·53‡
No	1068 (66·8%)	1064 (67·9%)	··	··
Yes	532 (33·2%)	503 (32·1%)	−0·01 (−0·05 to 0·03)‡	··
Overcorrection in study eye at 1 year	··	··	··	0·44§
No	1587 (99·2%)	1558 (99·4%)	··	
Yes	13 (0·8%)	9 (0·6%)	0·00 (−0·01 to 0·00)§	
Eyelid contour abnormality in study eye at 1 year	··	··	··	0·17‡
No	1542 (96·4%)	1525 (97·3%)	··	··
Yes	58 (3·6%)	42 (2·7%)	−0·01 (−0·02 to 0·00)‡	··
Lid closure defect in study eyes at 1 year	··	··	··	0·50†
No	1598 (99·9%)	1567 (100%)	··	··
Yes	2 (0·1%)	0	−0·00 (−0·00 to 0·00)*	··
Granuloma in study eyes at 1 year	··	··	··	0·73†
No	1595 (99·7%)	1564 (99·8%)	··	··
Yes	5 (0·3%)	3 (0·2%)	−0·00 (0·00 to 0·00)*	··
Pain score in study eyes at 1 year¶	5·35 (1·5)	5·32 (1·4)	−0·03 (−0·14 to 0·09)‡	0·63‡
Intraocular pressure elevation ≥10 mm Hg from baseline in study eyes at week 4	··	··	··	0·58†
No	1612 (99·7%)	1577 (99·6%)	··	··
Yes	5 (0·3%)	7 (0·4%)	0·00 (−0·00 to 0·01)*	··
Intraocular pressure elevation ≥10 mm Hg from baseline in study eyes within 1 year	··	··	··	1·00†
No	1609 (99·0%)	1577 (99·0%)	··	··
Yes	16 (1·0%)	16 (1·0%)	0·00 (−0·01 to 0·01)*	··
Any incidence of cataract surgery in study eyes by 1 year	··	··	··	0·34†
No	1618 (99·6%)	1590 (99·8%)	··	··
Yes	7 (0·4%)	3 (0·2%)	−0·00 (−0·01 to 0·00)*	··
Any incidence of adverse events attributed to study treatment by 1 year∥	··	··	··	0·27†
No	1195 (99·3%)	1197 (99·7%)	··	··
Yes to not serious	9 (0·7%)	4 (0·3%)	−0·00 (−0·01 to 0·00)*	··

Data are n (%), adjusted risk difference (95% CI), p value, or mean (SD). PTT=postoperative trachomatous trichiasis. logMAR=log(minimal angle of resolution), defined as log10(visual acuity fraction). *From exact method for calculating 95% CI of risk difference. †From Fisher’s exact test. The corresponding risk difference was not adjusted for inter-eye correlation and surgeon due to the small number of events. ‡From generalised regression models that account for inter-eye correlation and are adjusted by the surgeon (the stratification factor randomisation). §From generalised regression models that account for inter-eye correlation without adjusting for surgeon due to the small number of events. ¶Pain scale score ranges from 5 (least pain) to 20 (most pain). ∣Adverse events were analysed at the participant level.

**Table 4:** Comparison of participant-reported outcomes at 1 year between treatment groups

	Placebo (n=1600 study eyes)	Fluorometholone (n=1567 study eyes)	Adjusted difference (95% CI)	p value
Degree of satisfaction with the trichiasis surgery outcome in study eyes at 1 year	··	··	··	0·04*
Very satisfied	1461 (91·3%)	1468 (93·7%)	0·02 (0·00 to 0·04)*	··
Satisfied	128 (8·0%)	93 (5·9%)	··	··
Neither satisfied nor dissatisfied	5 (0·3%)	4 (0·3%)	··	··
Dissatisfied	5 (0·3%)	2 (0·1%)	··	··
Very dissatisfied	1 (0·1%)	0	··	··
Degree of satisfaction with the cosmetic outcome in study eyes at 1 year	··	··	··	0·12*
Very satisfied	1458 (91·1%)	1457 (93·0%)	0·01 (0·00 to 0·03)*	··
Satisfied	136 (8·5%)	105 (6·7%)	··	··
Neither satisfied nor dissatisfied	3 (0·2%)	4 (0·3%)	··	··
Dissatisfied	2 (0·1%)	1 (0·1%)	··	··
Very dissatisfied	1 (0·1%)	0	··	··
Scale of satisfaction with the outcome of trachomatous trichiasis surgery at 1 year†	9·69 (0·84)	9·71 (0·74)	0·02 (−0·04 to 0·09)‡	0·46‡
Health utility by EQ-5D at 1 year§				
Severity index (0–25, higher is worse)	6·33 (2·61)	6·32 (2·69)	−0·01 (−0·22 to 0·21)‡	0·96‡
Health state score (0–100, higher is better)	80·23 (15·51)	79·60 (15·66)	−0·67 (−1·91 to 0·56)‡	0·28‡

Data are n (%), adjusted risk difference (95% CI), p value, or mean (SD). *From generalised regression models that account for inter-eye correlation using generalised estimating equations and are adjusted by the surgeon (the stratification factor randomisation). †On a scale of 1–10 (a higher score indicates more satisfaction). ‡From linear regression models with adjustment by the surgeon (the stratification factor randomisation). §Measured at participant level instead of eye level.

## Data Availability

In accordance with National Institutes of Health and journal policies, a summary, de-identified data set, data analysis code, and data dictionary supporting the results in this publication will be made available by the Data Center by the time of publication at https://www.med.upenn.edu/cpob/flame. In addition, key derived variables will be contained in the datasets. The rights and privacy of people who participated in the FLAME trial will be protected at all times by stripping the data from all identifiers that could lead to disclosing the identity of individual research participants. The study’s protocol and statistical analysis plan are included in this publication as supplemental materials (also available at https://www.med.upenn.edu/cpob/flame). Release of data must be approved by the executive committee as long as it continues to meet. During this period, researchers can contact the principal investigator or Data Center chair to request limited-access datasets. After approval by the Executive Committee, the requesting investigator will need to enter into a data sharing agreement with the Data Center before obtaining data, after which the Data Center will share the data. Researchers requesting limited-access datasets will bear the cost of their preparation and will be responsible for following FLAME and National Eye Institute policy in their use. After the executive committee officially ceases FLAME trial operations, data supporting this paper can be accessed from the data repository established by the study put on file with the Center for Preventive Ophthalmology and Biostatistics data repository housed at the University of Pennsylvania Perelman School of Medicine (https://www.med.upenn.edu/cpob/flame). Current information regarding how to request data supporting the results of this analysis (via posted contact information for the study chair and Data Center director while the FLAME Research Group remains operational, or by download afterward) will be posted at https://www.med.upenn.edu/cpob/flame.

## Research in context

### Evidence before this study

We undertook an evidence-based review of OVID MEDLINE, Embase, Web of Science Core Collection, and Cochrane Library to evaluate what is known about the impact of fluorometholone on the incidence of postoperative trachomatous trichiasis (PTT), mediated through a reduction in postoperative inflammation. We searched articles published from database inception to Sept 8, 2025, using the terms “trichiasis” and “fluorometholone” as words in the title, abstract, or keyword list without limits on the year or language of publication. We used the broader term trichiasis rather than trachomatous trichiasis or PTT to make the search highly sensitive to finding relevant papers. The search yielded 42 references before de-duplication, and 29 after duplicates were removed. Manual review of the references by two coauthors (JHK and AAMo) found that the only original data evaluation of the question was from our previous publication evaluating three different doses of fluorometholone 0·1% suspension in a phase 2-like, dose-varying randomised clinical trial. That previous study reported that all three doses of fluorometholone were associated with about 30% lower incidence of PTT than seen with placebo (artificial tears) or in contralateral eyes. The present study was a randomised, controlled clinical trial including 2410 participants with 12 months of follow-up which, however, was designed as a preliminary look into the strategy without sufficient sample size to show a statistically significant difference in the incidence of PTT. Both this study and a broader evidence-based review about strategies to reduce the incidence of PTT called for a more definitive evaluation of fluorometholone as a potentially scalable strategy to reduce PTT incidence.

### Added value of this study

The Fluorometholone as Adjunctive Medical Therapy for Trachomatous Trichiasis (FLAME) trial was a large-scale, field-based, individually randomised, double-masked clinical trial sponsored by the National Eye Institute of the National Institutes of Health. The trial enrolled 2410 patients (sufficient to detect a 25% reduction in PTT with 90% power and type I error probability of 5%) and randomly allocated them (1:1) to fluorometholone 0·1% suspension or placebo (artificial tears) twice daily for 28 days. We selected the lowest of the three doses from the preliminary trial to minimise treatment risk, given that all three doses studied appeared to have similar benefit. To balance the known important individual factor of surgeon capability, randomisation was stratified by surgeon with block sizes of two and four, to ensure balanced allocation. There was a very high 98% follow-up rate throughout 12 months of follow-up. For the primary endpoint of cumulative incidence of PTT by 12 months, the odds ratio was 1·00 (95% CI 0·80·1–25). Thus, the study achieved its goal in showing that fluorometholone did not decrease the incidence of PTT by 25% or more, the level we hypothesised might motivate use of the treatment at a programmatic level. Given inefficacy, non-use of fluorometholone dominated in health economic analysis. However, fluorometholone twice daily for 28 days was not associated with increased incidence of adverse events.

### Implications of all the available evidence

The results provide convincing evidence that fluorometholone 0·1% suspension twice daily for 28 days is safe but not efficacious or cost-effective for reducing the incidence of PTT.
