# Supplementary material for: Evaluation of fluorometholone as adjunctive medical therapy for trachomatous trichiasis surgery (FLAME): a parallel, double-blind, randomised controlled field trial in the Jimma Zone, Ethiopia
Source: Lancet Glob Health. Author manuscript; Available in PMC 2026 Mar 30. (PMC13034013; doi:10.1016/S2214-109X(25)00493-0)
Supplement: 1 [file NIHMS2150010-supplement-1.pdf]

THE LANCET
Global Health

Supplementary appendix 1

This translation in Amharic was submitted by the authors and we reproduce it as supplied. It has not been peer reviewed. *The Lancet*'s editorial processes have only been applied to the original in English, which should serve as reference for this manuscript.

ይህ የአማርኛ ትርጉም ተመራማሪዎቹ ባዘጋጁት መሰረት የቀረበ እና በሌላ ወገን ያልተገመገመ መሆኑን እናስገነዝባለን። በላንሴት የአርትዖት ሂደቶች የተገመገመው በእንግሊዝኛ ቋንቋ የቀረበው ጽሑፍ ብቻ በመሆኑ ለዚህ ጽሑፍ ማጣቀሻ ሆኖ ማገልገል ያለበት በእንግሊዝኛ የተዘጋጀ መሆኑን እናሳስባለን።

Supplement to: Kempen JH, Abashawl A, Mohammed AA, et al. Evaluation of fluorometholone as adjunctive medical therapy for trachomatous trichiasis surgery (FLAME): a parallel, double-blind, randomised controlled field trial in the Jimma Zone, Ethiopia. *Lancet Glob Health* 2026; published online Jan 12. https://doi.org/10.1016/S2214-109X(25)00493-0.

በዓይን ማዝ (ትራኮማ) በሽታ ሳቢያ የሚከሰት የዓይን ሽፋሽፍት (ጠጉር) ወደ ውስጥ መቀልበስ (Trachomatous Trichiasis) ቀዶ ሕክምና፡ ፍሎሮሜታሎንን (fluorometholone) እንደማጠናከሪያ (ተጉዋዳኝ) ሕክምና አጠቃቀም በተመለከት (FLAME)፡ በጂማ ዞን፡ ኢትዮጵያ ውስጥ የተከናወነ መመዘኛ የመስክ ጥናት

John H Kempen, Aida Abashawl, Ahlam Awad Mohammed, Sarity Dodson, Wondu Alemayehu, Fangming Jin, Alemu Gemechu, Aemero Abateneh Mengesha, Dereje Adugna Kumsa, Yineng Chen, Kathleen McWilliams, Berhanu Tulu, Genemo Abdela, Alemayehu Megersa, Tolossa Cheru, Gadisa Mohammad, Tony Succar, Vatinee Y Bunya, K Davina Frick, Maureen G Maguire, Matthew J Burton, Gui-Shuang Ying, FLAME Trial Research Group

አጠቃላይ መነሻ

የዓይን ማዝ በሽታ፡ የዓይን ሽፋሽፍትን ወደውስጥ በመቀልበስ የዓይን እይታ እክልን የማስከተል ጠንቅ/ምክንያት ነው። ይህንን ችግር ለማከም ከሚከናወነው የተቀለበሰውን ጠጉር የማቃናት የዓይን ቆብ ቀዶ ሕክምና በኋላ፡ በከፍተኛ ቁጥር፡ የማገርሸት (የሽፋሽፍቱ መቀልበስ /መመለስ/) ሁኔታ ይከሰታል። ስለዚህ፣ ከቀዶ ሕክምናው ጋር በአባሪነት በቀን ሁለት ጊዜ ለ 28 ቀናት የሚሰጥ ፍሎሮሜታሎን 0.1% (ከዚህ በኋላ ፍሎሮሜታሎን) በዓይን ጠብታ የፀረ-ብግነት (anti inflammatory) ሕክምና፡ ይህንን ከቀዶ ሕክምና በኋላ እንደገና የሚከሰት ማገርሸትን (postoperative trachomatous triciasis - PTT) ደህንነቱን በጠበቀ፤ ውጤታማ እና ወጪ ቆጣቢ በሆነ መንገድ ይቀንስዋል ብለን መላ ምት (hypothesis) ተመርኩዘን ተነሳን።

አሰራር

በዚህ ጥናት ኣስተማማኝና ሚዛናዊ በሆነ መንገድ መድሃኒት መሰል (placebo) ሰው ስራሽ የዓይን የዕንባ ጠብታን (artificial tears) ከፍሎሮሜታሎን ጠብታ ጋር የማነጻጸር ጥናት በገጠር በሚገኙ በኢትዮጵያ የጅማ ዞን የጤና ኬላዎችና ጤና ጣቢያዎች የቀዶ ሕክምና በተከናወነላቸው የ15 አመት እና ከዛ ዕድሜ በላይ በሆኑ ተሳታፊዎች ዓይኖች ላይ፡ ተከናውኗል።። ለተሳታፊዎቹ የመድሃኒት መሰሉና (placebo)እና የፍሎሮሜታሎን ምደባ ዕኩል በዕኩል (1:1) በዕጣ ላይ የተመሰረተ ሲሆን፡ ተሳታፊዎች በተመደቡበት ቡድን ውስጥ ሆነው የሚጨርሱበት ጥናት ነበር። የሕክምናውና የቀዶ ሕክምናው ሐኪምም ኣመዳደብ በኣግባቡ የተገደበ ነበር። ተሳታፊዎች፡ የቀዶ ሕክምና ባለሞያዎች፡ እና የውጤት ገምጋሚዎች፡ በየትኛው የመድሃኒት አይነት ቡድን እንደተመደቡ ለማወቅ የማያስችል ዘዴ (masking) ተተግብሯል። ውጤቶቹ በ4ኛ ሳምንት፤ በ6ኛ ወር እና በ12ኛ ወር የቀዶ ሕክምና ከተከናወነበት ቀን በሁዋላ ተገምግመዋል። ዋናው የምርምሩ ውጤት፣ አጠቃላይ የ12-ወር የግርሽቱ መጠን (PTT) ድምር ክስተት ነበር። የግርሽቱ ክስትተ መጠን ከሚከተሉት በአንዱ ይገለፃል፡ አንድ ወይም ከዚያ በላይ ሽፋሽፍቶች የዓይንን ኩዋስ ዉጫዊ ክፍል መንካት፤ የሽፋሽፍት መንቀል ምልክት ካለ፤ ወይም የድጋሚ ቀዶ ህከምና መከናወን መረጃ። ይህ ጥናት በዓለም አቀፍ ደረጃ በClinicalTrials.gov፣ NCT04149210 ተመዝግቧል።

ግኝቶች

ከነሐሴ 15፡ 2013 እስከ ኅዳር 21፡ 2017 ባለው ግዜ ፡ 2410 ተሳታፊዎች (1692 [70·2%] በጾታ ሴት እና 718ቱ [29·8%] ወንድ ሲሆኑ፡ 3235 ዓይኖች) ተመልምለዋል። 1204 ተሳታፊዎች በፍሎሮሜታሎን ቡድን እና 1206 በመድሃኒት መሰሉ ቡድን ውስጥ በዕጣ ተመድበው ነበር። 823 (34·1%) ተሳታፊዎች በሁለቱም የዓይን ቆቦች ላይ የሽፋሽፍት መቀልበስ ቀዶ ማስተካከያ ሕክምና ተደርጎላቸው ነበር። የሁለቱ ቡድን ተመዳቢ ተሳታፊዎች የመነሻ ሕክምና ባህሪያት ግኝቶች ተመሳሳይ ነበሩ። 1180 (98·0%) በፍሎሮሜታሎን እና 1181 (97·9%) በመድሃኒት መሰሉ ቡድን ውስጥ ተሳትፈው በ12ኛ ወር ላይ ግምገማ ተደርጎላቸዋል። በማከም ዕቅድ (intention to treat) ትንተና፣ የሽፋሽፍት መቀልበስ ማገርሸት ድምር ክስተቶች፡ 12 ወራት የክትትል ግዜ ውስጥ በመድሃኒት መሰል ቡድን 218 (13·4%) ከ1625 ዓይኖች፤ በፍሎሮሜታሎን ቡድን ደግሞ 213 (13·4%) ከ1593 ዓይኖች (95% Cl -2% እስከ 2% ልዩነት) ነበሩ። በቡድኖቹ መሀል በቅድሚያ የተለዩ/የተገለፁ/፡ የሁለተኛ ደረጃ ውጤታማነት እና የደህንነት መስፈርቶች፡ በስታቲስቲክስ የሚደገፍ ጥንካሬ ያለው ልዩነት አልነበራቸውም (ሁሉም $p \geq 0{\cdot}10$ ናቸው)። ከጥናቱ ሕክምና ጋር የተያያዘ የአሉታዊ ክስተት ምዘና፡ በመድሃኒት መሰሉ ቡድን ውስጥ ዘጠኝ (0·7%) እና በፍሎሮሜታሎን ቡድን አራት (0·3%) ነበር ($p = 0{\cdot}17$)። የእርካታ መጠኑ ደረጃ ደግሞ በቀዶ ሕክምናው (ረካ ወይም በጣም ረክቷል በሚለው) ለ3156 (99.7%) ከ3167 ዓይኖች ተመዝግቧል። ፍሎሮሜታሎን ውጤታማ ባለመሆኑ፡ የጤና ኢኮኖሚያዊ ግምገማ፡ በፕሮግራም መጠቀምን የሚደግፍ ሆኖ ኣልተገኘም።

ትንታኔ /ትርጓሜ/

ፍሎሮሜታሎንን በቀን ሁለት ጊዜ ለ4 ሳምንታት መጠቀም ደህንነቱ አስተማማኝ ሆኖ ተገኝቶ ነበር። ነገር ግን የሽፋሽፍት መቀልበስ ማገርሸትን በመቀነስ ረገድ ውጤታማ ወይም ወጪ ቆጣቢ አልነበረም። ስለዚህ፣ ይህንን ችግር ለማዳን ወይም ለመቋቋም በሚተገበረው ፕሮግራም ለመጠቀም አይመከርም።

የገንዘብ ድጋፍ
ብሔራዊ የዓይን ተቋም (ብሔራዊ የጤና ተቋማት) እና ኤቢቢቪ።
