# Supplementary material for: Evaluation of fluorometholone as adjunctive medical therapy for trachomatous trichiasis surgery (FLAME): a parallel, double-blind, randomised controlled field trial in the Jimma Zone, Ethiopia
Source: Lancet Glob Health. Author manuscript; Available in PMC 2026 Mar 30. (PMC13034013; doi:10.1016/S2214-109X(25)00493-0)
Supplement: 2 [file NIHMS2150010-supplement-2.pdf]

THE LANCET
Global Health

Supplementary appendix 2

This translation in Oromifa was submitted by the authors and we reproduce it as supplied. It has not been peer reviewed. *The Lancet*'s editorial processes have only been applied to the original in English, which should serve as reference for this manuscript.

Hiikni Oromiffaa kun kan barreessitootaan galee fi nutis kan baay'isnu akkuma dhufeen/dhihaateen. Barreessitootaan irra deebiin hin ilaalamne. Adeemsi gulaallii "Lancet" hojii irra kan oole barreefffama jalqabaa Ingliffaa kan barreeeffama kanaaf akka wabiitti tajaajiluu qabu qofa irratti.

Supplement to: Kempen JH, Abashawl A, Mohammed AA, et al. Evaluation of fluorometholone as adjunctive medical therapy for trachomatous trichiasis surgery (FLAME): a parallel, double-blind, randomised controlled field trial in the Jimma Zone, Ethiopia. *Lancet Glob Health* 2026; published online Jan 12. https://doi.org/10.1016/S2214-109X(25)00493-0.

Itoophiyaatti Godina Jimmaa keessatti Gamaaggama “fluorometholone” akka wal’aansa dabalata (deeggarsa) baqaqsanii yaaluu dhukkuba labooba ija keessatti biqilee: “a parallel, double-blind, randomised controlled field trial”

John H Kempen, Aida Abashawl, Ahlam Awad Mohammed, Sarity Dodson, Wondu Alemayehu, Fangming Jin, Alemu Gemechu, Aemero Abateneh Mengesha, Dereje Adugna Kumsa, Yineng Chen, Kathleen McWilliams, Berhanu Tulu, Genemo Abdela, Alemayehu Megersa, Tolossa Cheru, Gadisa Mohammad, Tony Succar, Vatinee Y Bunya, K Davina Frick, Maureen G Maguire, Matthew J Burton, Gui-Shuang Ying, FLAME Trial Research Group

Guduunfaa

Duubessa hibirkuu/tiraakoomaa: dhukkubni laboobaa ijaa keessoo ijaatti deebi’uu(trachomatous trichiasis) karaa tiraakoomaan agartuu ittiin dadhabsiisu dha. Baqaqsanii yaaluu laboobaa ijaa keessoo ijaatti deebi’ee erga baqaqsanii yaaluun taasifame booddee taateen deebi’ee dhufuu isaa akka malee ol’aanaadha. Yaadni qorannoo keenyaa kan jedhu dawaan dhiita’uu/mada’uu qaamaa “fluorometholone 0·1% suspension” (kanaan booddee **fluorometholone)** yaalii baqaqsanii yaaluu waqtii baqaqsanii yaaluu keessatti guyyaatti copha lama guyyoota 28f yoo itti cophsame balaas hingeessu, bu’a qabeessaa fi baasiinis madaalawaadha.

Tooftaalee qorannoo: Qorannoo “randomized, parallel, placebo-cotroled trial” kana keessatti hirmaattota waggaa 15 fi isaa olii kan ija isaanii yaalii baqaqsanii hodhuu qola ijaa taasifatan buufataalee fi kellaawwan fayyaa godina Jimmaa irratti dibata ija laaffisu “**artificial tears**” (placebo) fi dawaa dhiita’uu/mada’uu qaamaa (**fluorometholone**) walcinaa qabne. Hirmaattotni otoo itti hinyaadiin garee dawaa madaa/dhiita’uu qaamaa fayyisuu(fluorometholone) fi garee dhangala’aa ija laaffisuu(placebo)tti reeshoo 1:1n qoodamanii turan. Dawaan dhiita’uu/madaa’uu qaamaa (**fluorometholone**) fi dhangala’aan ija laaffisu otoo itti hinyaadiin garee-gareen ogeessa baqaqsanii yaaluutiif ni hirama. Hirmaattotni, ogeessi baqaqsanii yaaluu, fi qorattootni bu’aa dawaa qoratan gosa dawaa hirmaattotni fudhatanii adda baafatanii akka hinbeekne dhokfamee ture. Baqaqsanii yaaluu booda bu’aan kan madaalame/qoratame torbaan 4, guyaa 28, ji’a jahaa fi waggaa tokkotti. Bu’aan dhukkubni laboobaa deebi’ee dhufuu kan qoratame cuunfaa waliigalaa deebi’ee dhufuu dhukkuba laboobaa ji’a 12 irratti argame: kan ittiin ibsamu yoo laboobaan tokko ykn isaa ol ija tuqaa jiraate; ragaa funaanamuu laboobaa agarsiisu; ykn yoo baqaqsanii yaaluun

irra deebiin godhameera ta’e dha. Qorannoon kun kan ittiin galmaa’e” ClinicalTrials.gov, NCT04149210” dha.

Argannoowwan: Gidduu Hagayya 19,2021 fi Sadaasa 30, 2024tti hirmaattotni 2410 (dhalaa 1692[70.2%], dhiira 718(29.8%); fi ijji 3235 qorannichaaf filamaniiru. Hirmaattotni 1204 garee **fluorometholone** keessattii fi hirmaattotni 1206 immoo garee **placebo** keessatti ramadamaniiru**.** Hirmaattotni 823(34.1%) baqaqsanii yaalu qola ija isaanii lameenuu kan godhatani dha. Ka’uumsa irratti amallii garee hirmaattotaa walfakkaataa ture**;** hirmaattotni 1180 (98.0%) garee fluorometholone fi 1181(97.9%) garee placebo dhuma ji’a 12 irratti xiinxalamanii turan. Xiinxala hirmaattota qorannoo hunda hammate (Intention-to-treat analysis) keessatti cuunfaan waliigala yeroo hordoffii ji’oota 12 laboobaan deebi’ee biqile gareen Placebo 218(13.4%) ijaan immoo ija 1625 yoo ta’u; garee **fluorometholone** keessaa hirmaattota 213(13.4%) fi ija 1593 (95% CI garaagarummaa -2% hanga 2%) ture. Garaagarumman bu’aa qabeessummaa fi nageenyummaan lammaffaa kan dura murtaa’e gareelee lameenuu gidduu hamma kana hunda hinqabu (not statistically different between groups: all $p \geq 0\cdot10$). Taateen faallaa yaalii qorannootiin walqabatee dhufe garee **“Placebo”** keessatti 0.7% fi garee **“fluorometholone”** keessatti immoo 0.3% ($p=0\cdot17$). Itti gammachuun yaala baqaqsanii yaaluu(itti gammadeera ykn baayyee itti gammadeera) hirmaattota 3156(99.7%) ijaan immoo ija 3167f galmaa’ee ture. Bu’a dhabeessummaa isaa irratti hundaa’uun, sagantaa baqaqsanii yaaluu qola ijaa keessatti hinfilatamu; kanaaf qusannaan baasii yaala fayyaa hubannoo keessa hingalle.

Hiikkaa: Deebi’ee dhufuu dhukkuba keessatti biqiluu laboobaa ijaa (PTT) hir’isuuf Fluorometholone’n guyyaatti si’a lama torbaan 4f yoo ijatti naqame/ namme miidhaa hinqabu; garuu baasii yaalaaf bahu hir’isuu fi bu’aa egamu fiduu irratti faayidaa hinqabu. Kanaaf, sagantaa dhukkuba Traakoomaa(Hibirkuu) dhabamsiisuu keessatti akka hojii irra ooluuf hingorfamu.

Deggarsa maallaqaa: National Eye Institute (National Institutes of Health) fi AbbVie
