# Supplementary material for: Evaluation of fluorometholone as adjunctive medical therapy for trachomatous trichiasis surgery (FLAME): a parallel, double-blind, randomised controlled field trial in the Jimma Zone, Ethiopia
Source: Lancet Glob Health. Author manuscript; Available in PMC 2026 Mar 30. (PMC13034013; doi:10.1016/S2214-109X(25)00493-0)

THE LANCET
Global Health

Supplementary appendix 1

This translation in Amharic was submitted by the authors and we reproduce it as supplied. It has not been peer reviewed. *The Lancet*'s editorial processes have only been applied to the original in English, which should serve as reference for this manuscript.

ይህ የአማርኛ ትርጉም ተመራማሪዎቹ ባዘጋጁት መሰረት የቀረበ እና በሌላ ወገን ያልተገመገመ መሆኑን እናስገነዝባለን። በላንሴት የአርትዖት ሂደቶች የተገመገመው በእንግሊዝኛ ቋንቋ የቀረበው ጽሑፍ ብቻ በመሆኑ ለዚህ ጽሑፍ ማጣቀሻ ሆኖ ማገልገል ያለበት በእንግሊዝኛ የተዘጋጀ መሆኑን እናሳስባለን።

Supplement to: Kempen JH, Abashawl A, Mohammed AA, et al. Evaluation of fluorometholone as adjunctive medical therapy for trachomatous trichiasis surgery (FLAME): a parallel, double-blind, randomised controlled field trial in the Jimma Zone, Ethiopia. *Lancet Glob Health* 2026; published online Jan 12. https://doi.org/10.1016/S2214-109X(25)00493-0.

Online Table 1: Characteristic of Surgeons by surgery type

TT Surgery Type Performed	Surgeon ID	Count of TT surgeries done Pre-FLAME	Number of surgeries/year Pre-FLAME	Number of years performing TT surgery pre-FLAME	Number of TT Surgeries Performed	Date of First TT Surgery for FLAME	Date of Last TT Surgery for FLAME	Time Performing Surgery in FLAME (Months)
**BLTR**	3001	1000	270	5·94	69	2021-09-01	2022-03-18	6·6
	3003	950	210	5·97	223	2021-08-19	2023-07-12	23·1
	3004	371	297	7·1	172	2021-08-19	2023-04-12	20
	3005	512	117	7·53	88	2023-02-13	2023-10-27	8·5
	3007	745	148	6·03	122	2021-09-01	2023-03-17	18·7
	3011	500	50	7·29	21	2021-10-28	2021-11-19	0·7
	3018	1959	300	7·05	77	2022-10-12	2023-10-06	12
**PLTR**	3001	1000	270	5·94	4	2022-03-11	2022-03-16	0·2
	3006	436	317	0·07	150	2021-08-20	2023-08-22	24·4
	3008	309	160	0·15	216	2021-08-19	2023-11-07	27
	3009	62	62	0·2	118	2021-09-02	2022-11-22	14·9
	3010	57	12	0·2	8	2021-09-02	2021-09-15	0·4
	3012	40	70	0·37	23	2021-11-02	2023-11-01	24·3
	3013	31	31	0·11	35	2022-01-01	2022-01-28	0·9
	3014	98	86	0·72	60	2022-01-02	2023-09-22	20·9
	3015	44	38	0·25	15	2022-03-16	2023-03-10	12
	3016	16	16	0·21	17	2022-03-07	2022-03-18	0·4
	3017	450	250	0·14	253	2022-01-18	2023-11-08	22
	3019	324	296	1·01	19	2022-10-17	2023-07-12	8·9
	3020	14	0	0·02	142	2022-11-23	2023-11-03	11·5
	3021	70	45	0·02	103	2022-12-21	2023-11-07	10·7
	3022	43	43	0·04	135	2022-12-22	2023-11-08	10·7
	3023	145	47	1·33	69	2023-02-13	2023-10-16	8·2
	3024	39	20	0·04	12	2023-02-15	2023-02-24	0·3
	3025	62	150	0·93	53	2023-02-17	2023-07-10	4·8
	3026	110	40	0·11	83	2023-02-20	2023-11-07	8.7
	3027	75	23	0·53	22	2023-06-20	2023-08-31	2·4
	3028	60	155	0·53	44	2023-06-21	2023-10-06	3·6
	3029	365	208	1·71	3	2023-08-24	2023-08-25	0
	3030	323	111	1·77	40	2023-09-19	2023-10-30	1·4
	3031	39	20	0·71	9	2023-09-20	2023-09-25	0·2

Online Table 2: Descriptive Analysis Results for Various Measures of Treatment Adherence of Participants at Week 4 by Randomized Treatment Groups Separately and Combined.*

	**Placebo** (N=1195 participants with Week 4 follow-up)	**Fluorometholone** (N=1189 participants with Week 4 follow-up)	Overall
**Distribution of Self-rated Adherence of using eye drops in study eyes (eye-level)**			
Number of study eyes	1617	1584	3201
Very good	1118 (69·14%)	1103 (69·63%)	2221 (69·38%)
Good	432 (26·72%)	400 (25·25%)	832 (25·99%)
Moderate	42 (2·60%)	40 (2·53%)	82 (2·56%)
Bad	21 (1·30%)	23 (1·45%)	44 (1·37%)
Very bad	2 (0·12%)	14 (0·88%)	16 (0·50%)
Not use	2 (0·12%)	4 (0·25%)	6 (0·19%)
**Percent of Expected Adherence based on bottle weight change (participant-level)**			
# of participants with bottle weight change data	932	935	1867
Mean (SD)	91·1 (43·3)	98·7 (46·4)	94·9 (45·0)
Median (Q1, Q3)	81·3 (63·0, 107·6)	90·7 (68·2, 118·8)	85·9 (65·6, 114·0)
Min, Max	12·1, 262·2	13·1, 306·8	12·1, 306·8
<25%	10 (1·07%)	12 (1·28%)	22 (1·18%)
25-50%	109 (11·70%)	85 (9·09%)	194 (10·39%)
>50-75%	272 (29·18%)	197 (21·07%)	469 (25·12%)
>75%	541 (58·05%)	641 (68·56%)	1182 (63·31%)
**Percent of Expected Adherence based on medication diary (participant-level)**			
# of participants with medication diary data	964	973	1937
Mean (SD)	84·7 (23·8)	84·62 (23·4)	84·7 (23·6)
Median (Q1, Q3)	96.3 (80.0, 100.0)	94.8 (79.3, 100.0)	95.5 (79.6, 100.0)
Min, Max	1·6, 125·0	0·0, 116·7	0·0, 125·0
<25%	32 (3·32%)	35 (3·60%)	67 (3·46%)
25-50%	93 (9·65%)	95 (9·76%)	188 (9·71%)
>50-75%	92 (9·54%)	99 (10·17%)	191 (9·86%)
>75%	747 (77·49%)	744 (76·46%)	1491 (76·97%)

*100=expected adherence. SD=standard deviation; Q1, Q3=lower and upper quartiles. Note that for each adherence measure data were less than complete, e.g. some participants did not bring their medication bottle or medication diary to the follow-up visit.

Online Table 3: Subgroup Analysis by sex for the comparison of incidence of PTT anytime during one-year follow-up§

	**Placebo (n=1,625 study eyes)**		**Fluorometholone (n=1,593 study eyes)**		**Adjusted risk difference (95% CI)***	**Adjusted odds ratios (95% CI)***	**P-value***
	**# of eyes**	**PTT (%)**	**# of eyes**	**PTT (%)**	..	..	..
**Sex**							
Female	1185	167 (14·1)	1127	150 (13·3)	-0·01 (-0·04, 0·02)	0·92 (0·71, 1·20)	Interaction P=0·24
Male	440	51 (11·6)	466	63 (13·5)	0·02 (-0·02, 0·07)	1·24 (0·80, 1·93)	

CI=confidence interval; PTT=Postoperative trachomatous trichiasis.

*From generalized regression models that account for inter-eye correlation and are adjusted by the surgeon (the stratification factor randomization), using the placebo group as the reference group.

§This subgroup analysis was not pre-specified, but was requested by the reviewer. The subgroup analysis was performed by calculating cumulative one-year incidence rate of PTT in placebo group and fluorometholone group, the adjusted difference between two treatment groups and adjusted odds ratio using placebo group as the reference in each subgroup, and the p-value for testing of interaction between treatment group indicator and subgroup indicator.

Supplemental Figure: Kaplan-Meier curves for the cumulative incidence of postoperative trachomatous trichiasisduring follow-up by treatment groups

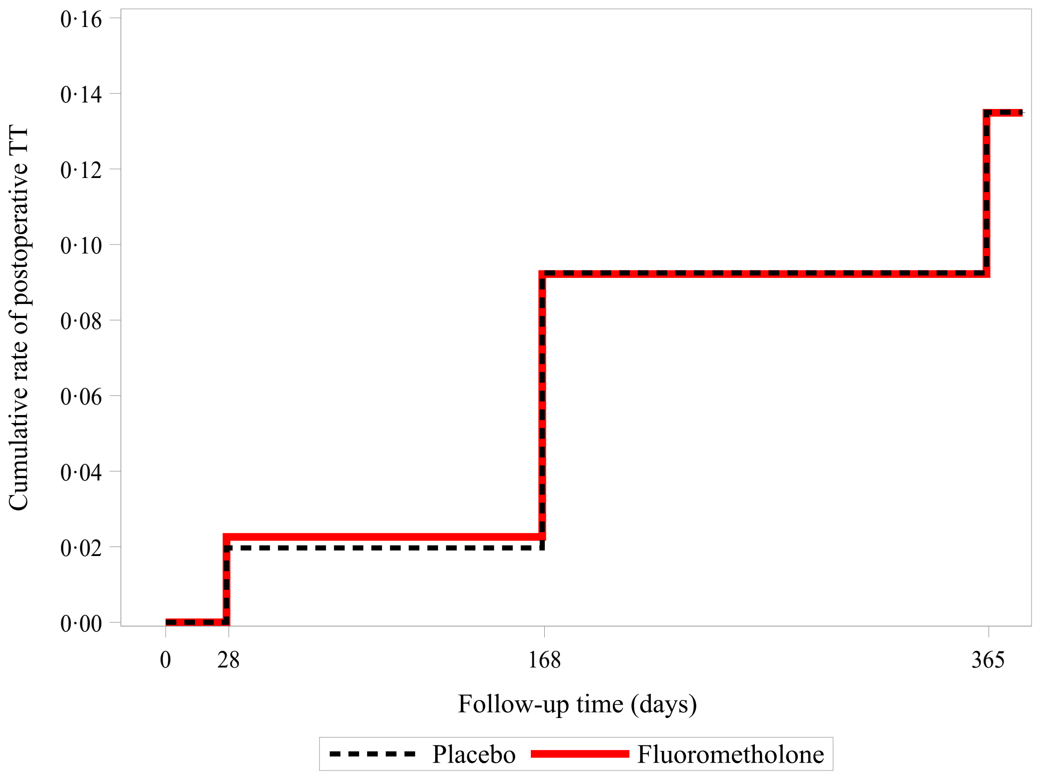

Supplement: 3 [file NIHMS2150010-supplement-3.pdf]
